# Effect of yeast probiotic *Saccharomyces cerevisiae* on the gut health of dogs undergoing rapid dietary transition

**DOI:** 10.3389/fmicb.2025.1561660

**Published:** 2025-05-15

**Authors:** Jia Xu, Chaoyu Wen, Guangming Song, Achraf Adib Lesaux, Hao Zhang, Yu Luo

**Affiliations:** ^1^Department of Veterinary Medicine, Faculty of Agriculture, Jinhua University and Vocational Technology, Jinhua, China; ^2^Haite Pet Nutrition & Health Institute, Hangzhou, China; ^3^Lesaffre Management (Shanghai) Co., Ltd., Shanghai, China; ^4^Phileo by Lesaffre, Marcq-en-Baroeul, France

**Keywords:** canine, gut microbiota, metabolomics, *Saccharomyces cerevisiae*, probiotics

## Abstract

**Introduction:**

Rapid dietary transition in dogs can disturb the balance of gut microbiota, resulting in symptoms such as diarrhea and compromised immune function. Yeast probiotic (*Saccharomyces cerevisiae*), has been shown to improve intestinal microbial composition and stimulate immune function. This study aims to investigate the effects of yeast probiotic *Saccharomyces cerevisiae* supplementation on hematology, serum biochemistry, fecal IgA, microbiota, and metabolites in dogs undergoing rapid dietary transition.

**Methods:**

Twenty healthy adult dogs were allocated into two groups: the control group (CON) and the yeast probiotic supplementation (YPS). All dogs were initially fed Diet 1 for 4 weeks, followed by an abrupt switch to Diet 2 for another 4 weeks. Throughout the study, the YPS group received 0.1% Actisaf^®^ Sc 50 product in a capsule given with their food, while the control group received a placebo.

**Results:**

Compared to the CON group, the YPS group exhibited lower serum globulin levels and higher albumin-to-globulin ratios on days 28 and 56 (*p* < 0.05). On day 56, the YPS group showed lower white blood cell counts and lower serum glucose levels (*p* < 0.05). Fecal IgA concentrations were higher in the YPS group on days 28, 30, 42, and 56 (*p* < 0.05). In the CON group, the abundance of Firmicutes significantly increased and the abundance of Fusobacteriota and Bacteroidota significantly decreased on days 42 compared to day 28 (*q* < 0.05). The YPS group showed a more stable gut microbiota transition post-dietary change. In the CON group, no significant changes in metabolite composition were observed 2 days after the dietary transition, but notable changes appeared after 2 weeks. In contrast, the YPS group exhibited substantial changes in metabolite composition 2 days after the diet change. Tyrosine metabolism showed significant changes in both groups of dogs following the dietary transition.

**Conclusion:**

*Saccharomyces cerevisiae* supplementation during rapid dietary transition in dogs led to beneficial changes in blood parameters, increased fecal IgA levels, and promoted a more stable gut microbiota. These findings suggest that yeast probiotics may support gut health and immune function during periods of dietary change.

## 1 Introduction

Intestinal microbiota plays a crucial role in dog health by stimulating the immune system, aiding metabolism, defending against pathogens, and providing nutritional benefits to the host (Mondo et al., 2019). Through fermentation of undigested nutrients in the hindgut, gut microbiota produce bioactive metabolites including short-chain fatty acids, secondary bile acids, and indole derivatives (Lyu et al., 2025). However, the composition of gut microbiota undergoes dynamic changes in response to various factors including age, diet, medication, and environmental conditions (Shah et al., 2024). Dysbiosis, or microbial imbalance in the gut, has been associated with various intestinal diseases in dogs, such as inflammatory bowel disease, as well as systematic diseases, such as obesity (Shah et al., 2024).

Dietary transition is a common practice in pet nutrition, often necessitated by life-stage changes, health conditions, product availability, or preference. Diet composition serves as a major determinant of microbial composition, as nutrients provide substrates for microbial fermentation (Pilla and Suchodolski, 2021). Although the canine gut microbiota demonstrates resilience and can eventually re-establish homeostasis following disturbances (Fassarella et al., 2021), rapid dietary changes can overwhelm this adaptive capacity. This may result in gastrointestinal discomfort, including diarrhea and compromised immune function (Wernimont et al., 2020). Therefore, gradual diet transitions, typically over a seven-day period, are recommended to minimize the risk of gastrointestinal disturbances. Nevertheless, abrupt dietary transitions can still occur due to practical constraints, potentially leading to microbial imbalances and gastrointestinal symptoms (Liao et al., 2023; Lin et al., 2020).

Probiotic supplementation has emerged a potential strategy to promote gut health during dietary transitions by modulating microbial composition and enhancing intestinal homeostasis (Ma et al., 2023). Yeast and its derivatives have been reported to provide certain benefits to dogs and cats due to their beneficial nutrients, such as proteins, vitamins, β-glucans, and mannooligosaccharides. However, research on live yeast in dogs and cats remains relatively limited (Maturana et al., 2023). Live yeast also exerts beneficial effects on intestinal health through stimulation of the immune system and suppression of pathogenic bacterial proliferation (Maturana et al., 2023). Furthermore, live yeast supplementation has been shown to mitigate gut microbiota disturbances caused by physiological stressor such as gestation and antibiotic administration in dogs (Arghavani et al., 2025; Garrigues et al., 2024). While previous research suggests that yeast may help stabilize gut health during sudden dietary transitions, comprehensive evaluations of its effects on systemic health markers, gut microbial composition, and immune responses remain limited (Bastos et al., 2023).

This study aimed to investigate the effects of yeast probiotic *Saccharomyces cerevisiae* supplementation on hematological and biochemical parameters, gut microbiota composition and metabolites through 16S rRNA sequencing and metabolomics, and fecal IgA concentrations in dogs undergoing rapid dietary transitions. The findings will provide deeper insights into the role of yeast probiotics in supporting gut health and immune function during periods of dietary stress.

## 2 Material and method

### 2.1 Animal and study design

All experimental procedures involving dogs were reviewed and approved by the Ethical Committee of Haite Pet Nutrition & Health Institute (EC0202D1), in accordance with Directive 2010/63/EU on the protection of animals used for scientific purposes.

Twenty healthy adult dogs were allocated into two groups: the control (CON) group and the yeast probiotic supplementation (YPS) group, each consisting of 10 dogs. The CON group included four beagles and six mongrels (six intact females and four intact males), with an average body weight (BW) of 8.65 ± 2.30 kg and an average of 1.7 ± 0.64 years; the YPS group included five beagles and five mongrels (four intact females and six intact males), with an average BW of 10.58 ± 2.89 and an average age of 2.1 ± 0.20 years. Dogs were housed individually but had access to an indoor enclosure for play and an outdoor playground in groups for at least 2 h daily. They were fed once daily, with the feeding amount calculated based on their body weight (Energy requirement = 132 × BW^0.75^) and adjusted weekly to maintain stable body weight. All dogs had water ad libitum. For adaption, all dogs were gradually switched to Diet 1 (Pedigree^®^ Dry Adult Dog Food for Small-Medium Breed Oral Health) over a four-day transition period during the first 2 weeks. They were then given Diet 1 exclusively for 4 weeks. On day 29, the dogs were abruptly switched to Diet 2 (Pro Plan^®^ Adult Weight Management Dry Dog Food) and continued this diet for 4 weeks. In the YPS group, the yeast probiotic Actisaf^®^ Sc (1 × 10^10^ CFU/g *Saccharomyces cerevisiae*, Phileo by Lesaffre, France) was administered at 0.1% of daily feed amount (1.18–3.28 × 10^9^ CFU/day) by adding one capsule to the dogs’ food throughout the study period, while the CON group received an empty capsule as a placebo. The study design and sample collection scheme are illustrated in Supplementary Figure 1.

### 2.2 Nutritional analysis of the diets

The crude protein, crude fat, crude fiber, and ash content of the diets were analyzed following Chinese standard methods (GB/T 6432-2018, GB/T 6433-2006, GB/T 6434-2022, and GB/T 6438-2007). The nutritional composition of the two diets is shown in Table 1. Feed samples for analysis was purchased and tested after the trial had concluded and were from a different batch than the diets used during the study.

**Table 1:** Guaranteed analysis on as is basis and ingredients list of the experimental diets.

Items	Diet 1	Diet 2
Crude protein, %	25.28	37.79
Crude fat, %	11.3	6.7
Crude fiber, %	2.1	9.7
Ash, %	6.8	6.5
Ingredients	Grains and their derivatives, meat and meat products (beef fat >3%), soybean oil, minerals, beet pulp (≥0.4%), peas (≥0.05%), glucose, xylose	Corn, corn gluten meal, pea husks, chicken meal, soy germ, soybean meal, barley, chicken liver digest, cellulose, chicken oil

Diet 1 is Pedigree^®^ Dry Adult Dog Food for Small-Medium Breed Oral Health; Diet 2 is Pro Plan^®^ Adult Weight Management Dry Dog Food.

### 2.3 Sample collection

Blood samples were collected from all dogs on day 0, 28, and 56 following an overnight fasting. Venous blood was drawn from the forelimb using a 0.7 × 25 mm needle. Samples were collected into tubes containing EDTA anticoagulant for immediate transport on ice packs at 4°C to the laboratory for hematology analysis. Additional samples were collected into non-anticoagulant tubes, allowed to clot at room temperature for 30 min, and then centrifuged at 3,500 *g* for 15 min to obtain serum for subsequent biochemical analysis.

Fecal samples were collected on day 0, 10, 27, 28, 30, 31, 42, and 56. Every day at 9 am, all feces excreted by the dogs from the previous day were collected in sealed bags and then stored at −20°C for subsequent determination of fecal dry matter (DM).

Fresh fecal samples were collected on day 0, 10, 27, 28, 30, 31, 42, and 56. Samples were obtained within 15 min of excretion using disposable sterilized sampling sticks. A portion of 3 g fresh fecal samples were immediately used for fecal pH testing, and the other portion of fresh feces were subdivided into three sterile fecal collection tubes, and then stored at −80°C for subsequent fecal IgA, fecal microbiota, and fecal targeted metabolites analysis.

### 2.4 Hematology and serum biochemistry

Blood collected in EDTA tubes was immediately analyzed for hematology using the Sysmex pocH-100i Analyzer (Sysmex Europe, Germany). Serum samples were subjected to biochemical analyzed using the IDEXX Catalyst One analyzer (IDEXX Laboratories, United States).

### 2.5 Fecal score, pH and DM

Fecal consistency was assessed using a 7-point scale method, with 1 to be very dry and 7 to be watery feces. Fecal pH was measured by diluting 3 g of fresh feces with 30 mL of distilled water and measuring with a portable digital pH meter (Hanna HI-98100, Italy). Fecal moisture was analyzed according to the GB/T 6435-2014. Fecal DM content was calculated as: Fecal dry matter content (%) = 100 − Fecal moisture (%).

### 2.6 Fecal IgA

One gram of fresh fecal sample was added to 9 mL of PBS at pH 7.2–7.4, and then thoroughly homogenized. The mixture was then centrifuged for about 20 min at 3,000 rpm, and the supernatant was carefully collected. Fecal IgA was detected using commercial canine ELISA kits (MM-2090O1, MEIMIAN, China).

### 2.7 Fecal microbiota

Genomic DNA was extracted from the samples using the Cetyl-trimethyl-ammonium bromide (CTAB) method. The concentration and purity of the DNA were assessed by electrophoresis on 1% agarose gels. DNA was then diluted to 1 ng/μL in sterile water, according to the measured concentration. The 16S rRNA gene fragment (16S V4) was amplified with specific primers (515F-806R) and a barcode. PCR reactions were performed with 15 μL of Phusion^®^ High-Fidelity PCR Master Mix (New England Biolabs), 2 μM of forward and reverse primers, and approximately 10 ng of template DNA. The thermal cycling conditions included an initial denaturation at 98°C for 1 min, followed by 30 cycles of 98°C for 10 s, 50°C for 30 s, and 72°C for 30 s, with a final extension at 72°C for 5 min. The PCR products were mixed with an equal volume of 1X loading buffer containing SYBR Green and subjected to electrophoresis on a 2% agarose gel. The PCR products were pooled in equidensity ratios and purified using the Qiagen Gel Extraction Kit (Qiagen, Germany). Sequencing libraries were prepared with the TruSeq^®^ DNA PCR-Free Sample Preparation Kit (Illumina, United States) following the manufacturer’s guidelines, and index codes were incorporated. Library quality was assessed using the Qubit^®^ 2.0 Fluorometer (Thermo Scientific) and the Agilent Bioanalyzer 2100. Finally, the library was sequenced on an Illumina NovaSeq platform, generating 250 bp paired-end reads.

Paired-end reads were assigned to samples based on unique barcodes, and barcodes and primer sequences were truncated. Raw tags were quality-filtered under specific conditions using the fastp (v0.22.0) to produce high-quality clean tags. Paired-end reads were merged using FLASH (v1.2.11). The tags were compared to the Silva 16S reference database^1^ using the UCHIME algorithm^2^ to identify and remove chimera sequences. The resulting clean tags were used for subsequent analysis. Sequence analysis was performed with Uparse software (v7.0.1001, http://drive5.com/uparse/), and sequences with ≥97% similarity were grouped into the same OTUs. A representative sequence for each OTU was selected for further annotation. Principal coordinate analysis (PCoA) was performed with the stats and ggplot2 packages in R software. Differential microbiota was identified using a *t*-test with false discovery rate (FDR) correction for *p*-values (*q*-value), and Linear Discriminant Analysis Effect Size (LEfSe) was conducted using LEfSe (v1.1.2) software.

### 2.8 Fecal targeted metabolites

Targeted metabolomics was employed to analyze free fatty acids, amino acids, bile acids, nucleotides, and related derivatives in fecal samples. Metabolite detection was carried out by a commercial laboratory (MetWare, Wuhan, China) using the AB Sciex QTRAP6500 LC-MS/MS platform. For sample preparation, 0.05 g of the sample was mixed with 500 μL of 70% methanol/water. The mixture was vortexed for 3 min at 2,500 rpm and then centrifuged at 12,000 rpm for 10 min at 4°C. A 300 μL of the supernatant was transferred to a new centrifuge tube and stored at −20°C for 30 min. The supernatant was centrifuged again at 12,000 rpm for 10 min at 4°C, and 200 μL of the final supernatant was collected for Protein Precipitation Plate processing before LC-MS analysis.

A standard curve was established by preparing standard solutions at different concentrations. The peak areas of the samples were then substituted into the standard curve equation to calculate the concentrations of the metabolites. Statistical analysis included false discovery rate (FDR) correction for *p*-values, resulting in *q*-values. Significantly metabolites between different time points and groups were identified based on VIP >1, |Log2FC| >1, and *q* < 0.05. VIP values were derived from orthogonal partial least squares discriminant analysis (OPLS-DA), which also provided score and permutation plots generated using the R package MetaboAnalystR. Data was log-transformed (log2) and mean-centered before performing OPLS-DA. To avoid overfitting, a permutation test (200 permutations) was conducted. An OPLS-DA model was considered reliable if *R*^2^*Y* and *Q*^2^ values exceeded 0.5, with *p*-values for both *Q*^2^ and *R*^2^*Y* being less than 0.05. After identifying the differential metabolites between days 30, 42, and 56 and the pre-diet transition baseline (day 28) in both the CON and YPS groups, pathway analysis was performed using MetaboAnalyst 6.0.^3^

### 2.9 Statistical analysis

All statistical analyses were performed using SPSS 26.0. Serum biochemistry and hematology data were analyzed using repeated measures analysis, with a Bonferroni *post-hoc* test to assess interactions between groups and time points. At each time point, fecal pH, DM, IgA, and differential metabolites between the CON group and the YPS group were tested for normality using the Shapiro–Wilk test. Data with a normal distribution were assessed using a *t*-test, while non-normally distributed data were analyzed using the Mann–Whitney *U* test. Differences in fecal scores between the CON and YPS groups at each time point were analyzed using the chi-square test. Statistical significance was set at *p* < 0.05.

## 3 Results

### 3.1 Serum biochemistry and hematology

The effects of diet and group on serum biochemical parameters were shown in Table 2. Significant interaction between time and group was observed on serum blood glucose, albumin, albumin/globulin ratio, and alkaline phosphatase (*p* < 0.05). Blood glucose levels in the CON group were significantly increased from day 0 to 56, whereas YPS group observed the opposite. Compared to CON group, YPS significantly increased albumin levels on day 56 and albumin/globulin ratio ratios on days 28 and 56 (*p* < 0.05). There was also a significant difference in globulin levels between CON and YPS groups (*p* < 0.05). However, except for the blood glucose concentration in the control group on day 0, which was below the reference range, all other serum biochemistry parameters were within the normal reference range.

**Table 2:** Effect of time and YPS on serum biochemistry in dog.

Items	Group	Time			*p*-value		
		Day 0	Day 28	Day 56	Time	Group	Time*Group
GLU	CON	67.10 ± 19.23^Bb^	94.70 ± 5.87^A^	93.60 ± 12.69^Aa^	0.003	0.717	0.003
	YPS	84.10 ± 11.87^Aa^	89.80 ± 9.87^A^	77.70 ± 12.52^Bb^			
CREA	CON	0.76 ± 0.11	0.77 ± 0.13	0.73 ± 0.15	0.349	0.886	0.376
	YPS	0.77 ± 0.12	0.73 ± 0.11	0.74 ± 0.05			
BUN	CON	15.50 ± 2.80	12.90 ± 2.02	15.60 ± 4.84	0.060	0.366	0.352
	YPS	16.70 ± 3.37	15.20 ± 3.55	15.40 ± 3.47			
BUN/CREA	CON	20.50 ± 1.96	17.60 ± 4.58	22.20 ± 8.47	0.288	0.486	0.156
	YPS	22.40 ± 4.86	21.40 ± 6.33	20.60 ± 5.23			
TP	CON	6.90 ± 0.27	6.62 ± 0.81	7.07 ± 0.51	0.024	0.142	0.337
	YPS	6.50 ± 0.26	6.59 ± 0.39	6.83 ± 0.16			
ALB	CON	3.29 ± 0.21^Aa^	2.99 ± 0.16^B^	3.12 ± 0.18^Ab^	0.010	0.955	<0.001
	YPS	2.99 ± 0.17^Bb^	3.15 ± 0.30^B^	3.34 ± 0.17^Aa^			
GLOB	CON	3.61 ± 0.34	3.82 ± 0.40^a^	3.94 ± 0.47^a^	0.197	0.005	0.341
	YPS	3.47 ± 0.24	3.44 ± 0.20^b^	3.51 ± 0.20^b^			
ALB/GLOB	CON	0.93 ± 0.13^A^	0.79 ± 0.08^Bb^	0.80 ± 0.11^Bb^	0.281	0.042	0.002
	YPS	0.88 ± 0.10	0.92 ± 0.10^a^	0.95 ± 0.10^a^			
ALT	CON	51.30 ± 12.16	45.60 ± 13.14	46.40 ± 16.55	0.718	0.146	0.147
	YPS	49.09 ± 11.97	55.80 ± 18.55	60.60 ± 18.19			
ALKP	CON	72.40 ± 38.06^B^	90.90 ± 60.53^A^	106.44 ± 68.43^A^	0.289	0.481	0.007
	YPS	84.60 ± 39.42	84.90 ± 34.37	69.60 ± 24.25			
CHOL	CON	194.20 ± 58.47	185.70 ± 52.20	161.00 ± 44.70	<0.001	0.314	0.497
	YPS	175.30 ± 41.39	174.20 ± 43.57	131.60 ± 40.18			
AMYL	CON	765.30 ± 126.86	763.20 ± 83.25	703.70 ± 106.33	0.196	0.941	0.250
	YPS	726.70 ± 133.74	773.10 ± 208.76	745.00 ± 141.14			
LIPA	CON	607.80 ± 305.60	504.30 ± 263.89	570.60 ± 304.70	0.533	0.769	0.177
	YPS	576.00 ± 220.33	604.40 ± 149.69	593.10 ± 188.07			
Ca	CON	9.83 ± 0.25	9.48 ± 0.50	9.51 ± 0.39	0.021	0.301	0.801
	YPS	9.61 ± 0.34	9.33 ± 0.53	9.44 ± 0.46			
P	CON	4.23 ± 0.61	4.08 ± 0.37	4.09 ± 0.64	0.116	0.765	0.268
	YPS	4.32 ± 0.56	4.34 ± 0.65	3.91 ± 0.35			

All data were expressed as mean ± SD. The uppercase letters A, B indicate significant difference between data in the same row. The lowercase letters a, b indicate significant difference between data in the same column. CON, the control group; YPS, the yeast probiotic supplementation group. GLU, blood glucose; CREA, creatinine; BUN, blood urea nitrogen; BUN/CREA, blood urea nitrogen/creatinine ratio; TP, total protein; ALB, albumin; GLOB, globulin; ALB/GLOB, albumin/globulin ratio; ALT, alanine aminotransferase; ALKP, alkaline phosphatase; CHOL, cholesterol; AMYL, amylase; LIPA, lipase; Ca, calcium; P, phosphorus.

As shown in Table 3, significant interactions between time and YPS were observed for white blood cell (WBC), mean corpuscular volume (MCV), mean corpuscular hemoglobin (MCH), eosinophil (EO), other lymphocyte, and mean corpuscular hemoglobin concentration (MCHC) in dogs (*p* < 0.05). Specifically, WBC, MCV, MCH, and EO levels on day 56 were significantly lower in the YPS group compared to day 0, whereas no significant changes were observed in the CON group. Conversely, MCH levels on day 28 were significantly lower in the CON group compared to day 0. It is important to note that although statistically significant, the observed changes, except for WBC, were numerically small. All hematology indices were within the normal reference range.

**Table 3:** Effect of time and YPS on hematology in dog.

Items	Group	Time			*p*-value		
		Day 0	Day 28	Day 56	Time	Group	Time*Group
WBC	CON	12.53 ± 2.52	11.89 ± 2.58	12.37 ± 2.77	0.017	0.789	0.023
	YPS	14.93 ± 4.09^A^	12.74 ± 2.97^AB^	9.74 ± 2.33^B^			
RBC	CON	7.02 ± 1.01	6.61 ± 0.87^b^	7.70 ± 1.57	0.238	0.194	0.196
	YPS	7.73 ± 1.39	7.44 ± 0.71^a^	7.44 ± 0.66			
HGB	CON	150.20 ± 19.97	139.40 ± 15.87^b^	164.10 ± 35.01	0.226	0.156	0.129
	YPS	167.00 ± 29.65	158.90 ± 17.10^a^	157.20 ± 14.56			
HCT	CON	0.45 ± 0.06	0.43 ± 0.05^b^	0.50 ± 0.10	0.292	0.077	0.077
	YPS	0.52 ± 0.09	0.48 ± 0.06^a^	0.48 ± 0.04			
MCV	CON	63.93 ± 3.30^b^	64.99 ± 2.24	65.08 ± 2.41	0.168	0.285	<0.001
	YPS	67.32 ± 2.37^Aa^	65.52 ± 2.42^B^	64.58 ± 2.38^B^			
MCH	CON	21.44 ± 0.64	21.12 ± 0.76	21.32 ± 0.83	0.002	0.836	0.048
	YPS	21.62 ± 0.83^A^	21.35 ± 0.86	21.12 ± 0.75^B^			
MCHC	CON	335.90 ± 13.73^Aa^	325.30 ± 4.55^B^	327.60 ± 8.00	0.511	0.096	0.004
	YPS	321.10 ± 7.00^b^	326.10 ± 7.22	327.40 ± 8.87			
PLT	CON	235.10 ± 117.87	275.40 ± 136.29	227.20 ± 144.45	0.125	0.914	0.392
	YPS	279.40 ± 152.89	263.10 ± 134.30	180.30 ± 107.22			
LYM%	CON	0.27 ± 0.07	0.26 ± 0.10	0.24 ± 0.09	0.652	0.418	0.069
	YPS	0.22 ± 0.08	0.21 ± 0.05	0.26 ± 0.07			
OTHR%	CON	0.66 ± 0.08	0.67 ± 0.11	0.69 ± 0.11	0.923	0.641	0.284
	YPS	0.69 ± 0.09	0.70 ± 0.06	0.68 ± 0.07			
EO%	CON	0.07 ± 0.02	0.08 ± 0.04	0.07 ± 0.04	0.099	0.410	0.349
	YPS	0.09 ± 0.03	0.09 ± 0.03	0.07 ± 0.01			
LYM	CON	3.35 ± 1.05	3.04 ± 1.48	2.89 ± 1.09	0.070	0.429	0.104
	YPS	3.15 ± 0.96	2.69 ± 0.79	2.49 ± 0.81			
OTHR	CON	8.30 ± 2.07	7.89 ± 2.30	8.67 ± 2.95	0.039	0.631	0.008
	YPS	10.54 ± 3.67^A^	9.01 ± 2.60^A^	6.61 ± 1.73^B^			
EO	CON	0.88 ± 0.38^b^	0.91 ± 0.40	0.81 ± 0.39	0.002	0.333	0.021
	YPS	1.24 ± 0.32^Aa^	1.04 ± 0.23^A^	0.64 ± 0.25^B^			
RDW-SD	CON	33.06 ± 2.12	34.68 ± 1.19	35.86 ± 1.18	<0.001	0.894	0.204
	YPS	33.97 ± 2.18	34.24 ± 1.75	35.67 ± 2.30			
RDW-CV	CON	0.12 ± 0.01	0.12 ± 0.01	0.13 ± 0.01	0.312	0.327	0.323
	YPS	0.11 ± 0.01	0.13 ± 0.01	0.72 ± 1.85			
PDW	CON	14.70 ± 0.35	13.69 ± 1.94	15.60 ± 1.47	0.001	0.723	0.072
	YPS	13.56 ± 1.40	14.81 ± 3.01	14.68 ± 1.24			
MPV	CON	12.11 ± 0.26^a^	11.64 ± 0.69	12.45 ± 0.70	0.009	0.442	0.519
	YPS	11.25 ± 0.41^b^	11.50 ± 1.13	11.68 ± 0.49			
P-LCR	CON	0.45 ± 0.02^a^	0.39 ± 0.075	0.49 ± 0.07	0.004	0.672	0.369
	YPS	0.37 ± 0.04^b^	0.39 ± 0.12	0.41 ± 0.05			

All data were expressed as mean ± SD. The uppercase letters A, B indicate significant difference between data in the same row. The lowercase letters a, b indicate significant difference between data in the same column. CON, the control group; YPS, the yeast probiotic supplementation group. WBC, white blood cell; RBC, red blood cell; HGB, hemoglobin; HCT, hematocrit; MCV, mean corpuscular volume; MCH, mean corpuscular hemoglobin; MCHC, mean corpuscular hemoglobin concentration; PLT, platelets; LYM%, lymphocyte percentage; OTHR%, other lymphocyte percentage; EO%, eosinophil percentage; LYM, lymphocyte; OTHR, other lymphocyte; EO, eosinophil; RDW-SD, red cell distribution width-standard deviation; RDW-CV, red cell distribution width-coefficient of variation; PDW, platelet distribution width; MPV, mean platelet distribution width; P-LCR, platelet large cell ratio.

### 3.2 Fecal characteristics

**Figure 1:**
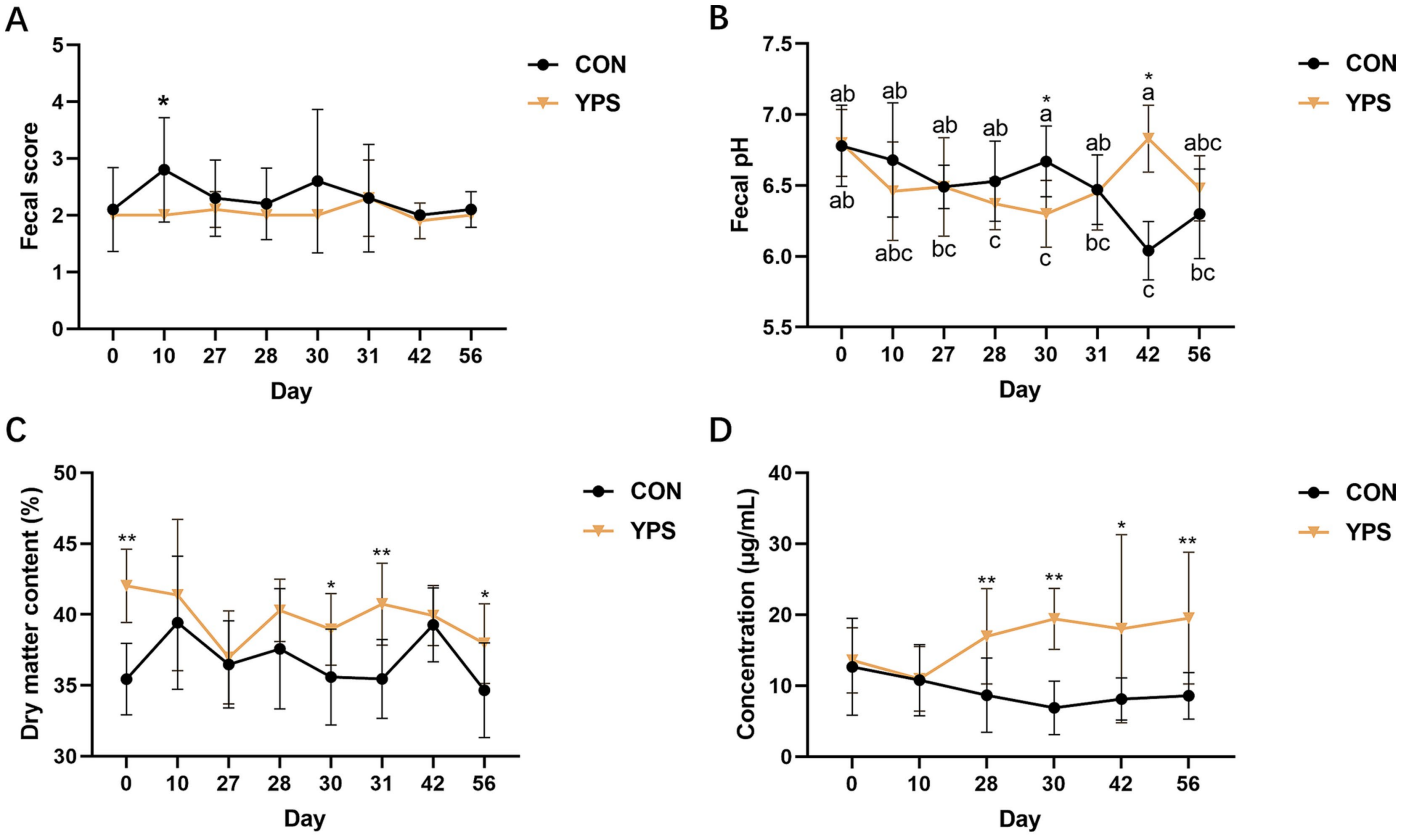
Effect of YPS on fecal score **(A)**, fecal pH **(B)**, fecal dry matter content **(C)**, and fecal IgA **(D)**. All data were expressed as mean ± SD. ^*^p < 0.05 and ^**^p < 0.01. CON, control group; YPS, yeast probiotic supplementation group.

The fecal characteristics is shown in Figure 1. During the diet transition period, none of the dogs experienced diarrhea. There was a significant increase in fecal scores in the CON group on day 10. YPS significantly decreased the fecal pH on day 30 compared to the CON group (*p* < 0.05). However, 2 weeks after dietary change, YPS significantly increased fecal pH (*p* < 0.05). Moreover, YPS significantly increased the fecal DM content on day 30, 31, and 54, but significant difference was also noted on day 0 (*p* < 0.05). The levels of fecal IgA were significantly increased in the YPS group on day 28, 30, 42, and 56 (*p* < 0.05).

### 3.3 Fecal microbiota

**Figure 2:**
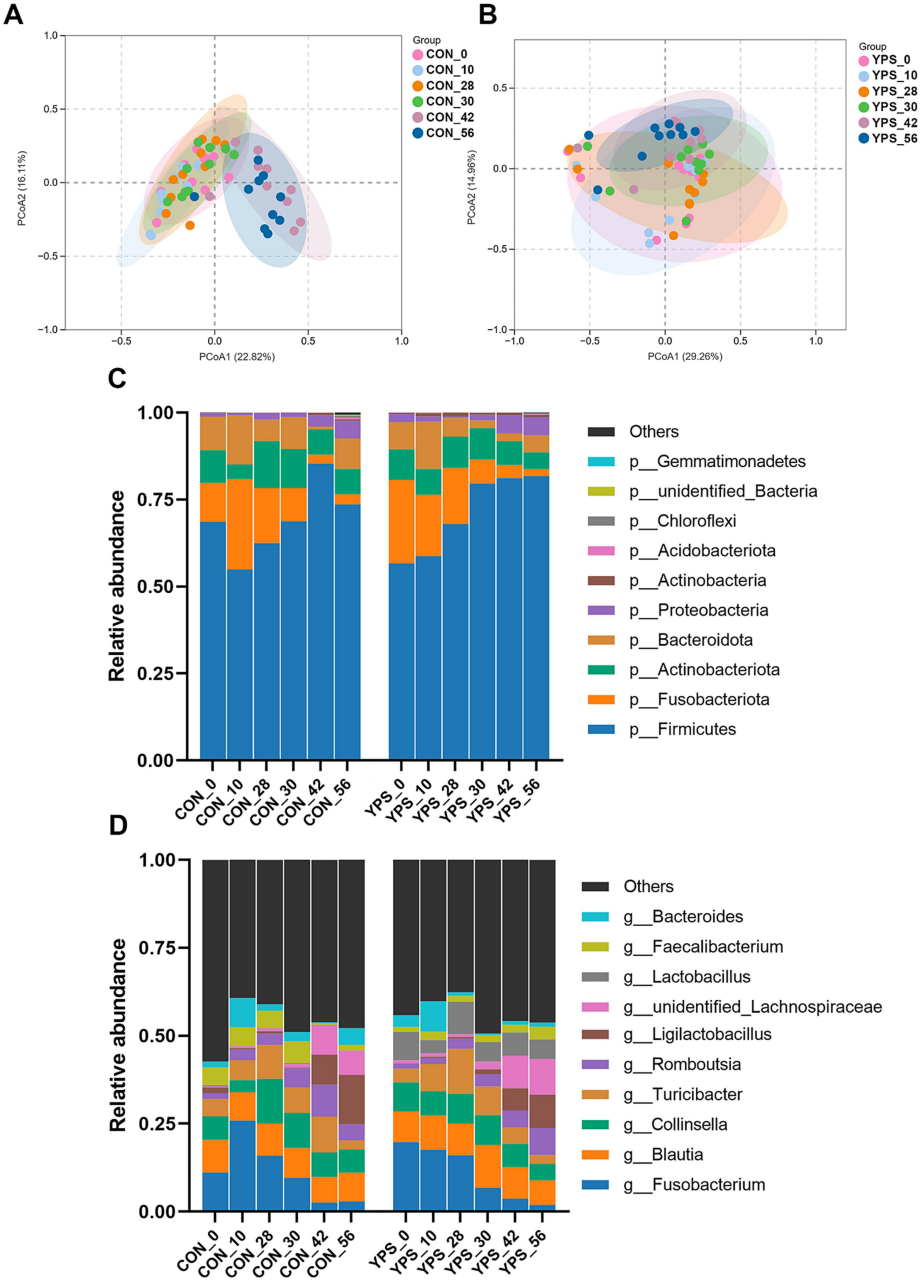
Diet alters the gut microbiota. The PCoA of CON **(A)** and YPS **(B)** group. Relative abundance of fecal microbiota at the phylum **(C)** and **(D)** genus levels. CON, control group; YPS, yeast probiotic supplementation.

**Figure 3:**
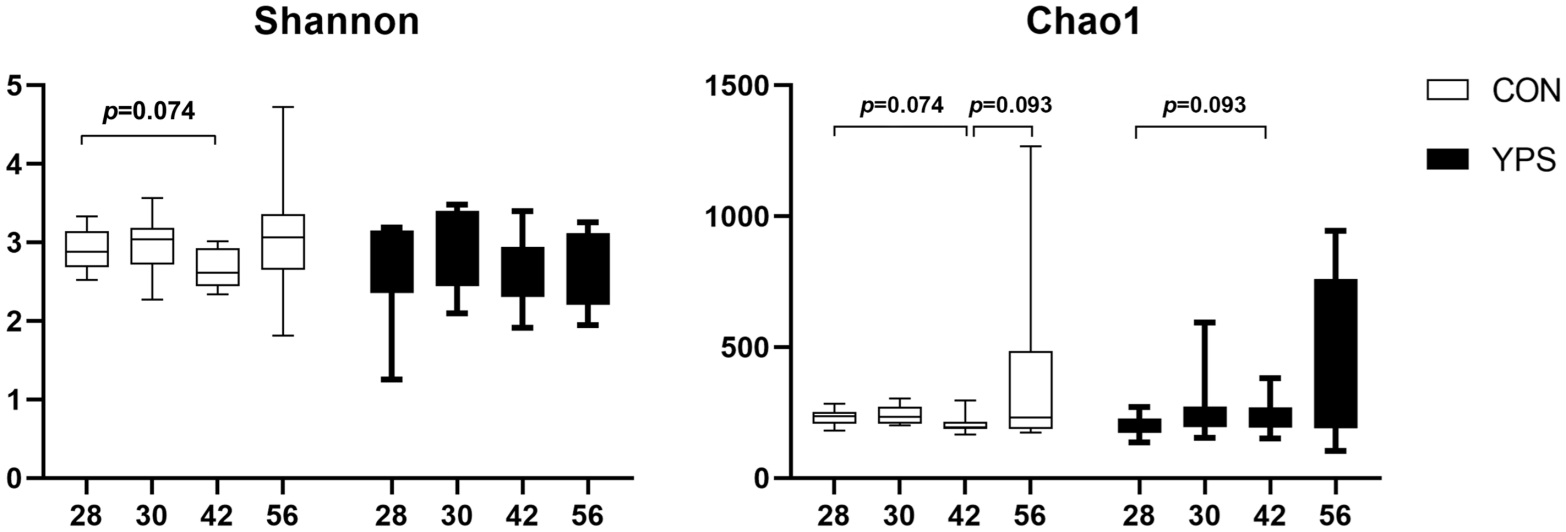
Change of alpha diversity after dietary transition. CON, control group; YPS, yeast probiotic supplementation group.

PCoA analysis revealed distinct differences in fecal microbiota composition between dogs fed different diets. In the CON group, microbiota composition remained consistent until day 30, immediately after the dietary transition, but began to show clear separation from day 42 (Figure 2A). In contrast, the YPS group displayed closer clustering of microbiota composition at different time points (Figures 2A,B). Detailed fecal microbiota composition at the phylum and genus levels at different time points are illustrated in Figures 2C,D. Significant shift in fecal microbiota composition over time were observed only in CON group, specifically between day 28 and day 42 (Supplementary Figure 2). On day 42, the CON group exhibited a notable increase in the abundance of Firmicutes (Supplementary Figure 2), accompanied by significant decreases in the abundance of Fusobacteriota and Bacteroidota significantly decreased at phylum level (*q* < 0.05). Alpha diversity analysis revealed variations in microbial community following dietary transition across groups (Figure 3). In the CON group, both the Shannon and Chao1 indices showed decreasing trends by day 42 compared to day 28, although Chao1 index showed an increasing trend from day 42 to day 56. In contrast, the YPS group exhibited an increasing trend in Chao1 index at day 42. However, no significant differences in alpha diversity were observed between the two groups throughout the study.

**Figure 4:**
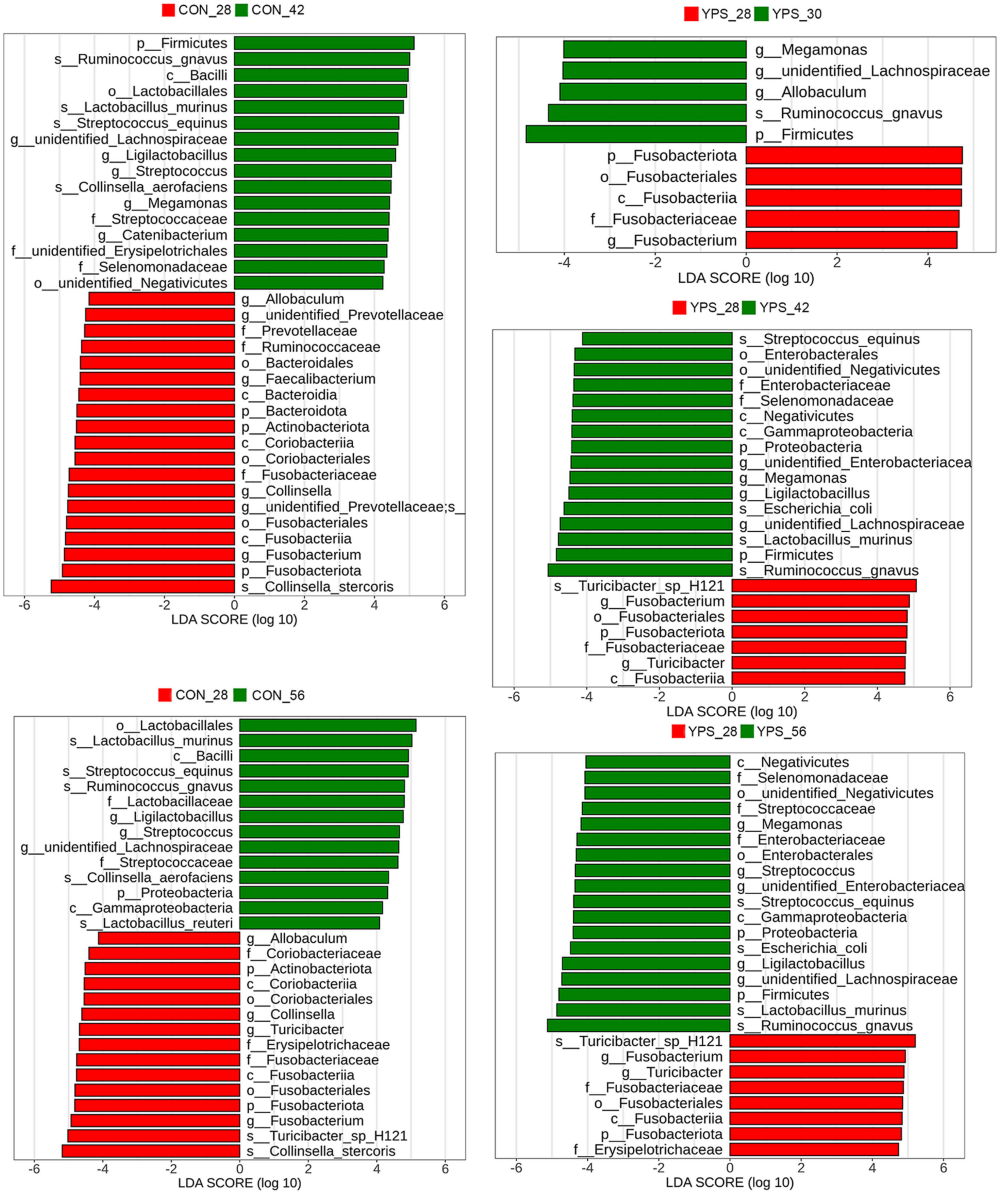
Fecal microbiota differences between day 28 and subsequent time points following rapid dietary transition in CON and YPS group. CON, control group; YPS, yeast probiotic supplementation.

Using day 28 as a baseline, LEfSe analysis was performed to identify microbial taxa that underwent significant changes post-diet transition (Figure 4). In the CON group, no significant changes were observed on day 30. However, by day 42, the relative abundances of *unidentified_Lachnospiraceae*, *Ligilactobacillus*, and *Streptococcus* were significantly higher, while the relative abundance of *Fusobacterium*, *Collinsella*, and *Allobaculum* were significantly lower. Moreover, the relative abundance of *Megamonas*, *Catenibacterium*, *Faecalibacterium*, and *unidentified_Prevotellaceae* showed significant changes only on day 42, while the relative abundance of *Turicibacter* significantly increased only on day 56. In the YPS group, the relative abundance of *Megamonas* and *unidentified_Lachnospiraceae* were significantly increased and the relative abundance of *Fusobacterium* was significantly reduced after dietary transition. The relative abundance of *Allobaculum* significantly increased only on day 30. The relative abundance of *unidentified_Enterobacteriaceae*, *Ligilactobacillus*, and *Turicibacter* showed significant changes between day 42 and day 28, as well as between day 56 and day 28, while the relative abundance of *Streptococcus* significantly increased only on day 56 (Figure 4).

### 3.4 Fecal metabolites

**Figure 5:**
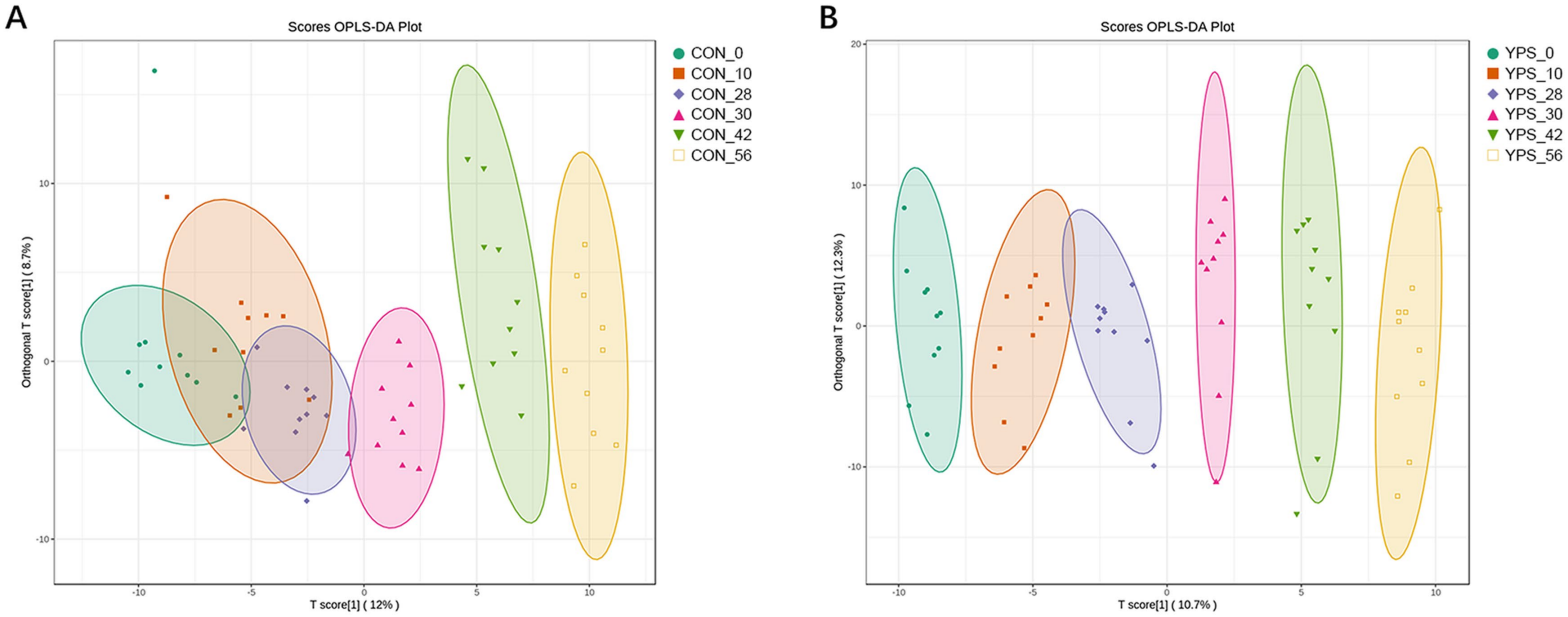
Diet alters the gut metabolite. The OPLSDA of CTR **(A)** and YPS **(B)** groups. CON, control group; YPS, yeast probiotic supplementation.

**Figure 6:**
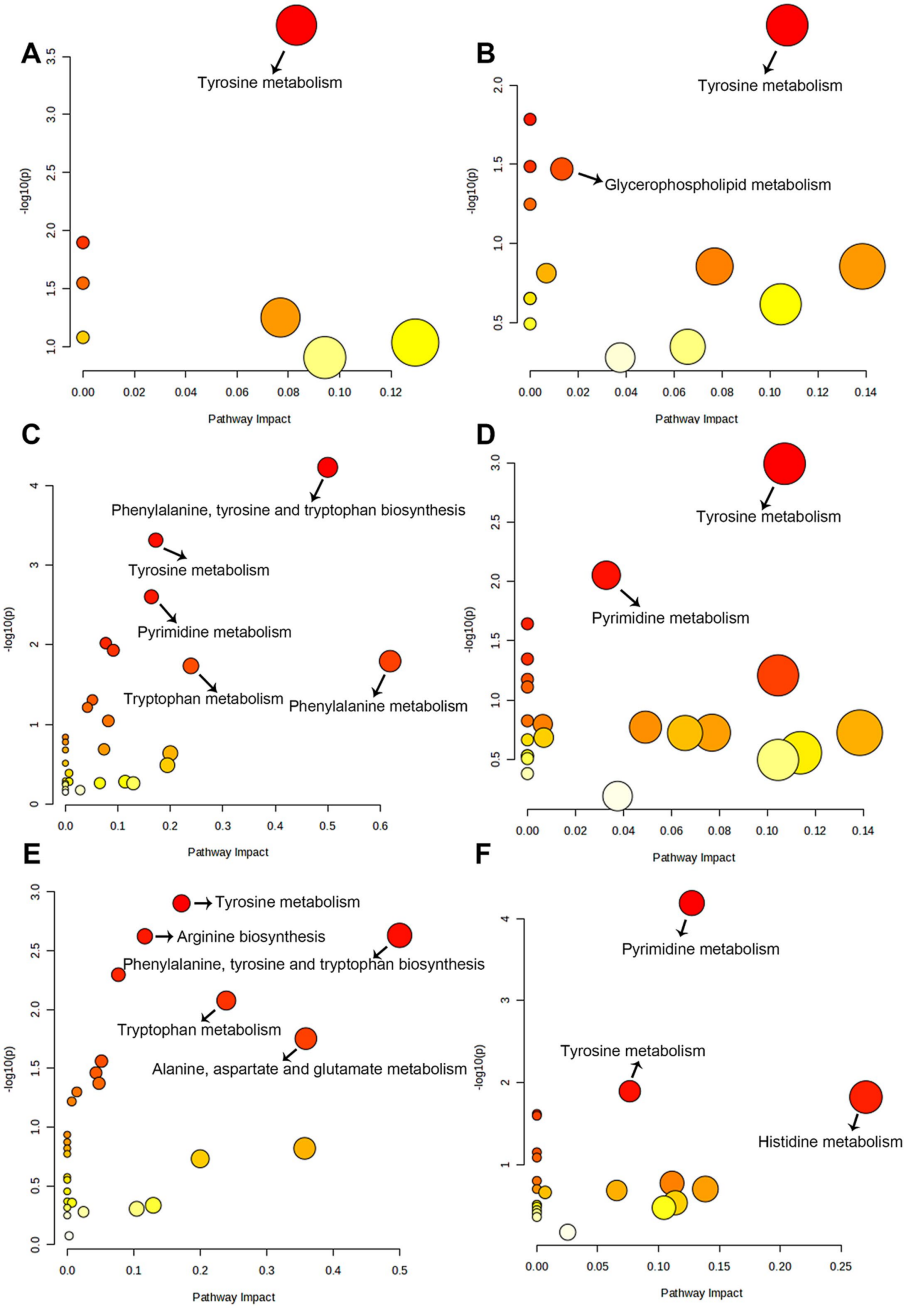
The pathways of significantly different metabolites between baseline (day 28) and day 30 **(A)**, 42 **(C)**, and 56 **(E)** in CON group. The metabolic pathways of significantly different metabolites between baseline (day 28) and day 30 **(B)**, 42 **(D)**, and 56 **(F)** in YPS group.

In total, 384 metabolites were identified. OPLS-DA (Figure 5) and PCA (Supplementary Figure 3) indicated a distinct fecal metabolites profile in YPS group as early as day 30 compared to the CON group. To assess metabolite changes following the diet transition, day 28 was used the baseline. Differential fecal metabolites were identified based on criteria |Log2FC| >1, VIP >1, and *q* < 0.05. In the CON group, the number of differential metabolites on days 30, 42, and 56 were 7, 97, and 84, respectively (Supplementary Figure 4A). In the YPS group, the number of differential metabolites on days 30, 42, and 56 were 33, 44, and 42, respectively (Supplementary Figure 4B). Pathway enrichment analysis via KEGG was used to identify the effects of diet transition on the metabolic pathways in dogs (Figure 6). In the CON group, significant changes were observed in tyrosine metabolism on day 30. On day 42, notable changes were observed in pathways including the phenylalanine, tyrosine and tryptophan biosynthesis; tyrosine metabolism; pyrimidine metabolism; phenylalanine metabolism; and tryptophan metabolism. On day 56, significant alterations were seen in tyrosine metabolism, arginine biosynthesis, phenylalanine, tyrosine, and tryptophan biosynthesis, tryptophan metabolism, and alanine, aspartate and glutamate metabolism. In the YPS group, significant changes were observed in tyrosine and glycerophospholipid metabolism on day 30, tyrosine and pyrimidine metabolism on day 42, and pyrimidine metabolism, tyrosine metabolism, and histidine metabolism on day 56.

**Figure 7:**
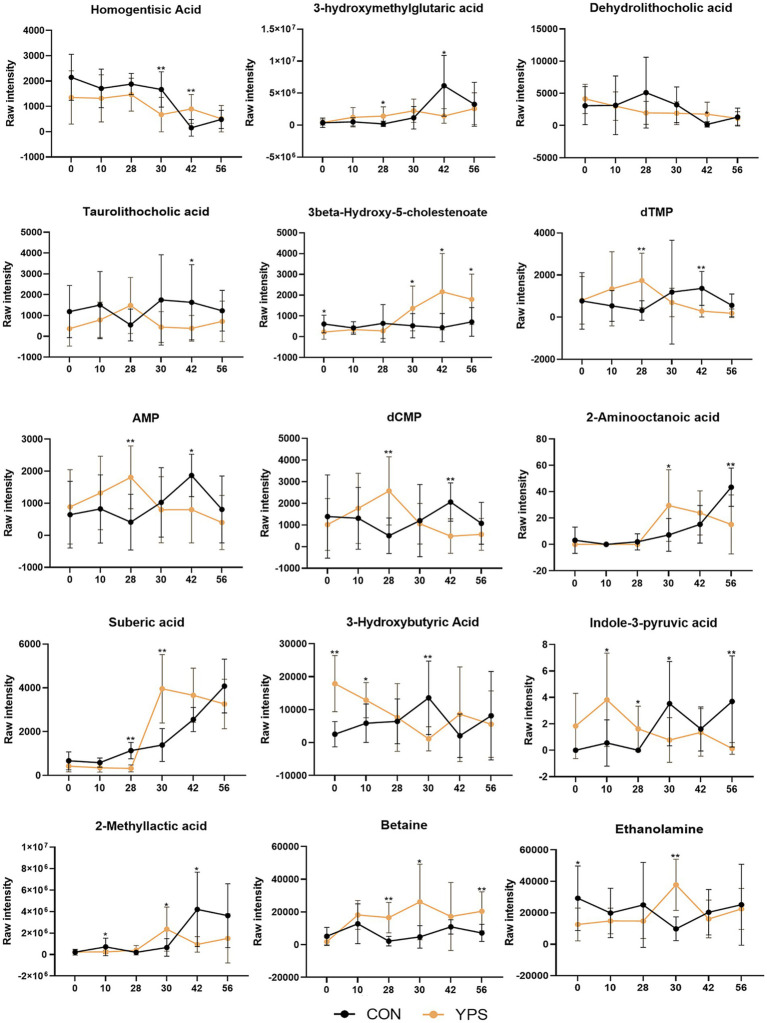
Differential metabolite changes between CON and YPS groups. All data were expressed as mean ± SD. ^*^p < 0.05 and ^**^p < 0.01. CON, control group; YPS, yeast probiotic supplementation group.

Differential metabolites between the CON group and the YPS group were also screened, there were 52, 25, and 6 significantly different metabolites on days 30, 42, and 56, respectively (Supplementary Figure 5). Of these, 15 metabolites exhibited notable shifts, initially showing higher or lower LogFC in the CON group but later shifting to higher or lower levels in the YPS group (the Log2FC values for YPS vs. CON shifted from negative to positive or from positive to negative). A total of 15 metabolites (Figure 7), including homogentisic acid, 3-hydroxymethylglutaric acid, dehydrolithocholic acid, taurolithocholic acid, 3beta-hydroxy-5-cholestenoate, dTMP, AMP, dCMP, 2-aminooctanoic acid, suberic acid, 3-hydroxybutyric acid, indole-3-pyruvic acid, 2-methyllactic acid, betaine, and ethanolamine.

**Figure 8:**
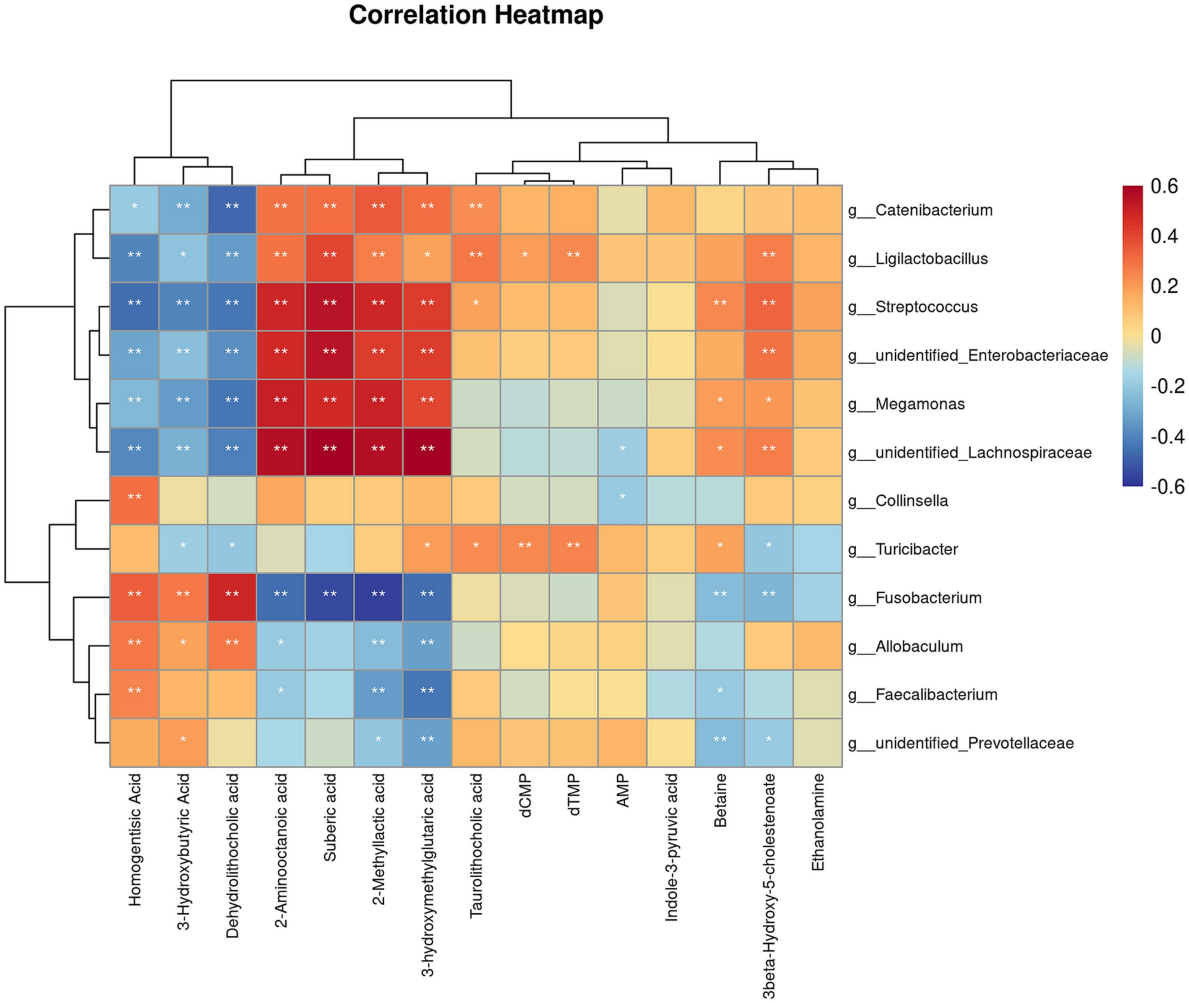
Correlation analysis of differential microorganisms and differential metabolites. The symbol (*) indicates a significant association between microorganisms and metabolites (*p < 0.05, and **p < 0.01).

Correlation analysis was performed to investigate the relationship between the changes in microbiota after the diet transition and the metabolites that exhibited a reversal in LogFC (Figure 8). We identified two distinct clusters in our analysis. The results showed that the abundances of *Catenibacterium*, *Ligilactobacillus*, *Streptococcus*, *unidentified_Enterobacteriaceae*, *Megamonas* and *unidentified_Lachnospiraceae*, which increased after the diet transition, were negatively correlated with homogentisic acid, 3-hydroxymethylglutaric acid, and dehydrolithocholic acid, and positively correlated with 2-aminooctanoic acid, suberic acid, 2-methyllactic acid, 3-hydroxybutyric acid and taurolithocholic acid. In contrast, the abundances of *Fusobacterium*, *Allobaculum*, *Faecalibacterium* and *unidentified_Prevotellaceae*, which decreased after the diet transition, were positively correlated with homogentisic acid, 3-hydroxymethylglutaric acid and dehydrolithocholic acid, and negatively correlated with 2-aminooctanoic acid, suberic acid, 2-methyllactic acid, 3-hydroxybutyric acid and taurolithocholic acid.

## 4 Discussion

Yeast probiotic, a rich source of proteins, minerals, and nucleic acids, is widely used as a functional food ingredient (Puligundla et al., 2020). Many studies have focused on yeast cell wall components, which contains prebiotics such as β-glucans and mannooligosaccharides known to stimulate the host immune system (Liu et al., 2021; de Oliveira Matheus et al., 2021; Soares et al., 2023; Kilburn-Kappeler and Aldrich, 2023). In contrast, live yeast has demonstrated a stronger ability to bind certain *Salmonella* strains *in vitro* compared to yeast cell wall only (Posadas et al., 2017). *Saccharomyces cerevisiae* may enhance gut microbiota by competing with pathogens for adhesion sites and by promoting the growth of beneficial bacteria (Sampath et al., 2023). Previous studies have found that yeast probiotic *Saccharomyces cerevisiae* reduces the dysbiosis index and enhances gut functionality during rapid dietary transitions in dogs (Bastos et al., 2023). This study further explores the effects of yeast *Saccharomyces cerevisiae* on gut metabolites and microbiota in dogs undergoing rapid diet transitions. In this study, we found an interaction effect between yeast and time on blood glucose levels, with a significant decrease observed in the yeast group at day 56. Previous reports have indicated that yeast may have hypoglycemic activity (Wu et al., 2021; Zhou et al., 2019). The high-viscosity β-glucan in yeast can slow gastric emptying and prolong intestinal transit time, thereby reducing glucose digestion and absorption (Bozbulut and Sanlier, 2019). The reduction in blood glucose may result from the combined effects of the high fiber content and yeast in Diet 2. A previous study also suggested that yeast probiotic seems to exhibit a greater capability to modulate the gut microbiota of dogs when the diet contains a higher fiber concentration (Bastos et al., 2023). Albumin levels are indicative of nutritional status, while globulin levels are markers of immunity and inflammation levels (Wu et al., 2019). Additionally, YPS significantly reduced serum alkaline phosphatase, which is commonly used for diagnosing liver diseases (Lowe et al., 2023). However, no abnormalities in blood glucose concentration were found in subsequent tests, this instance of hypoglycemia might be due to an extended fasting period. White blood cell serve as the body’s defense system against pathogens, and their count typically increases during infection or inflammation (Glenn and Armstrong, 2019). Decreased white blood cell in YPS group might indicate a potential modulation of immune response or a reduced inflammatory state associated with the supplementation.

Regarding fecal characteristics, we did not observe any symptoms of diarrhea in the dogs following the rapid diet transition. This may be due to the high-fiber diet improving the stool consistency to some extent. Fecal pH is typically considered to be related to the content of the short-chain fatty acids (Yamamura et al., 2023). Previous studies have found that rapid changes in diet can lead to a decrease in fecal pH in dogs (Liao et al., 2023; Lin et al., 2020), and the addition of prebiotics such as yeast cell-wall extract alone can also cause a decrease in fecal pH (Beloshapka et al., 2012). However, this study did not measure the levels of short-chain fatty acids. The impact of rapid dietary changes on fecal pH requires further investigation. Fecal IgA significantly increased after 28 days of yeast probiotic supplementation. IgA is the predominant antibody in the mucosal immune system, playing a crucial role in maintaining intestinal mucosal and microbial homeostasis by inhibiting microbial adhesion through immune exclusion mechanisms (Li et al., 2020). While a previous study suggested that increased microbial diversity due to yeast supplementation in neonatal dairy calves may be linked to enhanced IgA secretion (Rostoll Cangiano et al., 2023), no significant differences in alpha diversity between the CON and YPS groups in this study. Additionally, β-glucans in yeast can stimulate IgA secretion by interacting with receptors on immune cells (Tanioka et al., 2013).

Diet can rapidly alter the gut microbiota (David et al., 2014), and the changes in fecal microbiota and metabolites caused by a rapid diet transition in dogs tend to stabilize within a few days (Lin et al., 2022). In this study, the CON group exhibited a decreasing trend in alpha diversity by day 42 following dietary transition, while the YPS group showed an increasing trend. Alpha diversity is commonly used indicator to evaluate the diversity and stability of gut microbiota in human (Willis, 2019), with greater diversity generally linked to enhanced microbial resilience (Lozupone et al., 2012). The observed decline in diversity in the CON group suggests that dietary transition temporarily disrupted the gut microbiota, which appeared to recover by day 56. In contrast, supplementation with *Saccharomyces cerevisiae* may have facilitated a quicker recovery of microbial diversity following the dietary shift. At the phylum level, we found that the abundance of Firmicutes gradually increased, while the abundance of Fusobacteriota gradually decreased after dietary transition. Previous similar studies found that a rapid transition from a low-protein, low-fiber diet to a high-protein, high-fiber diet decreased the abundance of Firmicutes (Bastos et al., 2023). Firmicutes have the ability to degrade dietary fiber (Sun et al., 2023). Studies have shown that feeding dogs a high-fiber diet has increased the abundance of Firmicutes in feces (Martinez-Lopez et al., 2021; Middelbos et al., 2010). In our study, Diet 2 had a higher crude fiber content, which may have increased the abundance of Firmicutes. At the genus level, we observed similar changes in *Streptococcus*, *Fusobacterium*, *Ligilactobacillus*, and *Turicibacter* after the dietary transition in both groups of dogs. *Streptococcus* are opportunistic pathogens that can cause localized infection or septicemia in dog (Lamm et al., 2010). In this study, the abundance of *Streptococcus* increased after the dietary transition. However, similar studies (Bastos et al., 2023) found a decrease in their abundance after switching from a low-protein, low-fiber diet to a high-protein, high-fiber diet. In both studies, no clinical symptoms were observed in the dogs. Additionally, we observed significant differences in the *Streptococcus* content between the two studies, suggesting that diet may affect individuals with varying microbial compositions differently. In dogs fed a high-fiber diet, the abundance of *Fusobacterium* was decreased (Martinez-Lopez et al., 2021; Middelbos et al., 2010), which is in consistent with our findings. *Ligilactobacillus* is considered a probiotic with characteristics such as antioxidant properties and the production of bioactive metabolites (Yang et al., 2024). *Turicibacter* has been found to be associated with host lipid metabolism (Lynch et al., 2023), with its abundance increasing in dogs after weight loss. In this study, after switching to a diet with lower fat content, the abundance of *Turicibacter* decreased in both groups of dogs. Furthermore, we found that abundance of *Allobaculum* decreased in the CON group after the diet change, but increased in the YPS group by day 30. *Allobaculum* is considered to be negatively correlated with inflammation, insulin resistance, and obesity (Zheng et al., 2021). Studies have shown that the abundance of *Allobaculum* decreases in obese dogs and in dogs undergoing weight loss compared to lean dogs (Macedo et al., 2022). In this study, we found a negative correlation between *Allobaculum* and 3-hydroxybutyric acid. 3-Hydroxybutyric acid is a ketone body found in the large intestine, which can serve as a substrate for gut microbes to produce butyrate, providing an energy source for the microbiota (Satoh and Sasaki, 2024). *Allobaculum* may produce butyrate by utilizing 3-hydroxybutyric acid as a substrate. Currently, the effectiveness of butyrate in treating obesity remains controversial (van Deuren et al., 2022). In this study, the dogs’ energy intake was kept constant before and after the dietary transition, with food intake adjusted to maintain stable body weight. The relationship between *Allobaculum* and obesity thus requires further investigation. Studies have shown that the abundance of *Allobaculum* increased after transport stress in dogs supplemented with *Saccharomyces cerevisiae* fermentation product (Oba et al., 2023). Yeast supplementation may help alleviate stress by increasing the abundance of *Allobaculum*.

Significant changes in tyrosine metabolism were observed in both groups at each time point following the dietary transition. Tyrosine can be metabolized by gut microbiota into p-cresol, which is subsequently converted to p-cresyl sulfate-a known uremic toxin that may impair renal function (Gryp et al., 2017). However, specific tyrosine-related metabolites were not quantified. The alterations in tyrosine metabolism may be related to the higher protein content in Diet 2 but need to be further confirmed (Friedman, 2004). Additionally, enrichment of glycerophospholipid metabolism was noted in the YPS group on day 30. Previous research has reported that yeast supplementation can influence glycerophospholipid metabolism in the plasma of bitches before parturition (Garrigues et al., 2024). Glycerophospholipids are key components of cell membranes (Osawa et al., 2024), and their metabolism is significantly upregulated in *Saccharomyces cerevisiae* under hypoxic stress. This process may contribute to maintaining cell membrane stability under such conditions (Xia et al., 2019). It is possible that *Saccharomyces cerevisiae* contributes to gut homeostasis by consuming intestinal oxygen, thus promoting the growth of anaerobic bacteria. However, further research is needed to validate this assumption.

This study has some limitations. Feed samples were not collected during the trial, and the nutritional composition was assessed afterward using newly purchased samples, which may not fully reflect the diets used during the study. Moreover, divergent trends in fecal pH were observed between the groups at days 30 and 42, but fecal short-chain fatty acids were not detected in the metabolomics analysis. Future research should further explore the effects of dietary changes in dogs, particularly those involving increased fat and protein levels using larger sample sizes.

## 5 Conclusion

This study found that dietary transitions significantly alter the composition of gut microbiota and their metabolites in dogs. Additionally, supplementation of yeast probiotic *Saccharomyces cerevisiae* attenuates these changes. Yeast probiotic *Saccharomyces cerevisiae* can increase fecal IgA levels in dogs and modulate changes in gut microbiota and metabolites after dietary transitions. However, further research is needed to confirm these potential effects on a larger population using different diet’s composition.

## Data Availability

The datasets presented in this study can be found in online repositories. The names of the repository/repositories and accession number(s) can be found at: https://www.ncbi.nlm.nih.gov/, PRJNA1237525.

## Footnotes

^1^ https://www.arb-silva.de/

^2^ http://www.drive5.com/usearch/manual/uchime_algo.html

^3^ https://www.metaboanalyst.ca/
