# Supplementary material for: Effect of yeast probiotic Saccharomyces cerevisiae on the gut health of dogs undergoing rapid dietary transition
Source: Front Microbiol. 2025 May 15;16:1561660. doi: 10.3389/fmicb.2025.1561660 (PMC12119570; doi:10.3389/fmicb.2025.1561660)
Supplement: Supplementary file 1 [file Data_Sheet_1.docx]

Supplementary Material


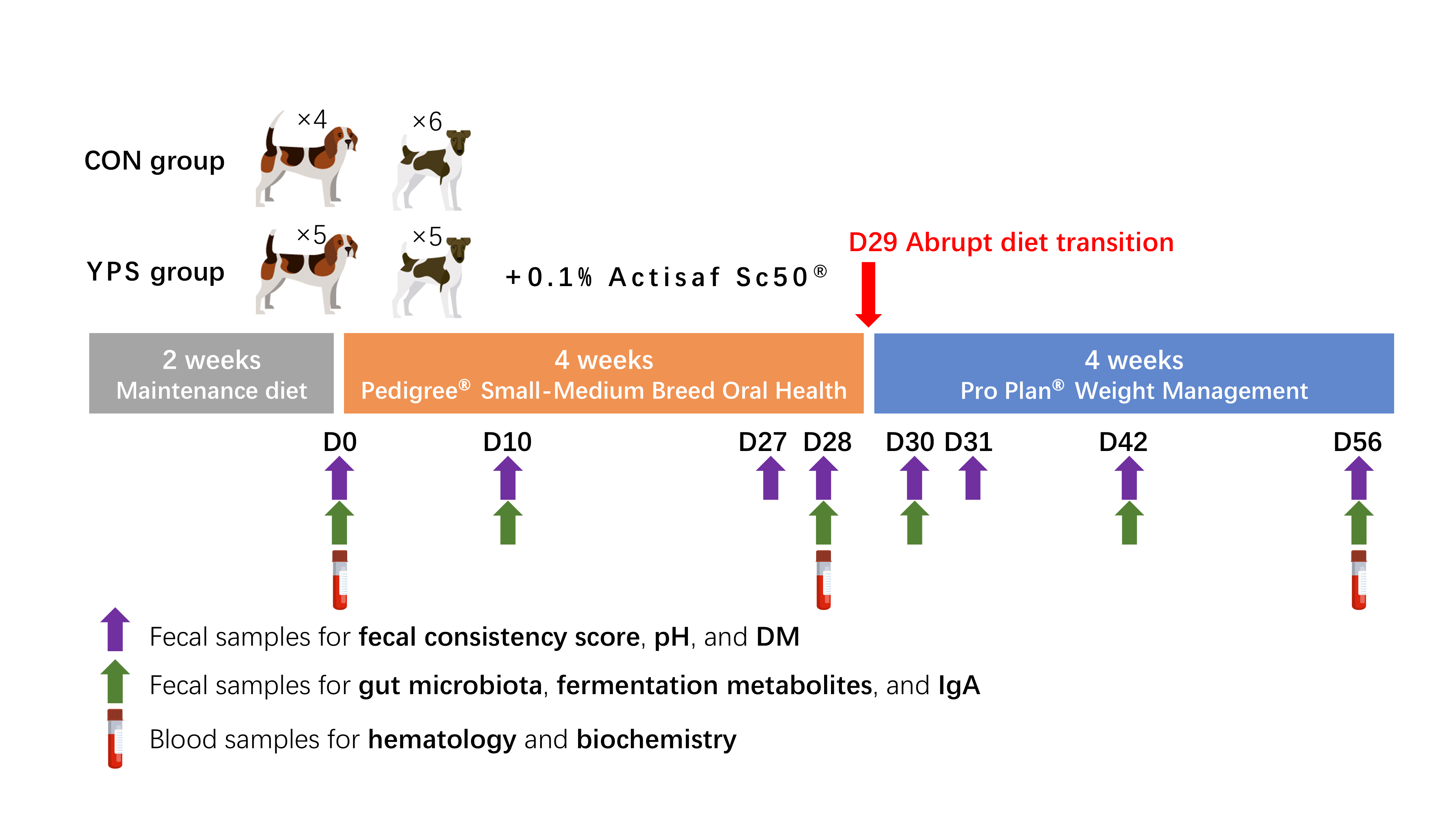


Supplementary Figure 1. The schematic diagram of the study design


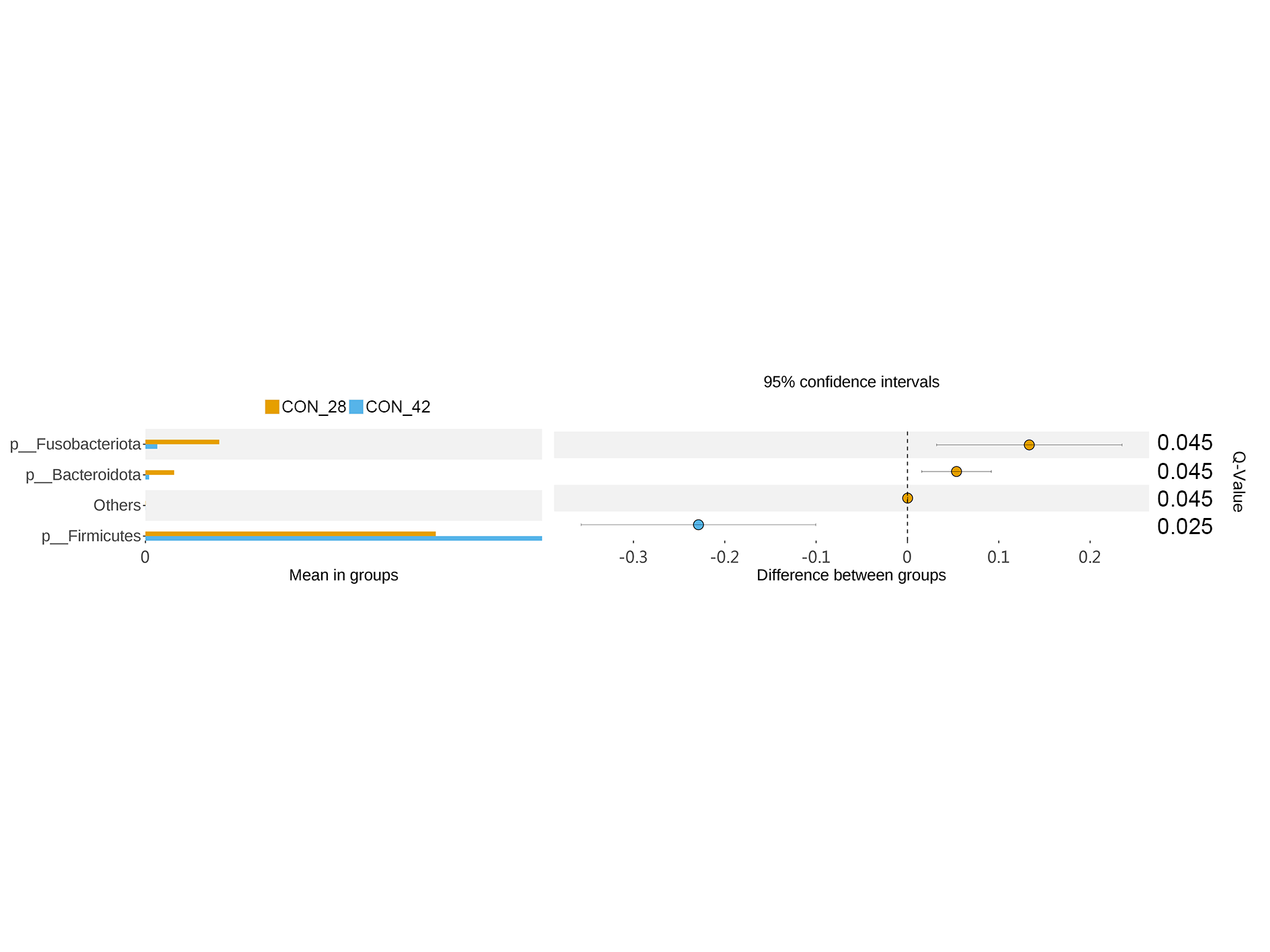


Supplementary Figure 2. The differential microbiota in the CON group between day 28 and day 42. CON, control group.


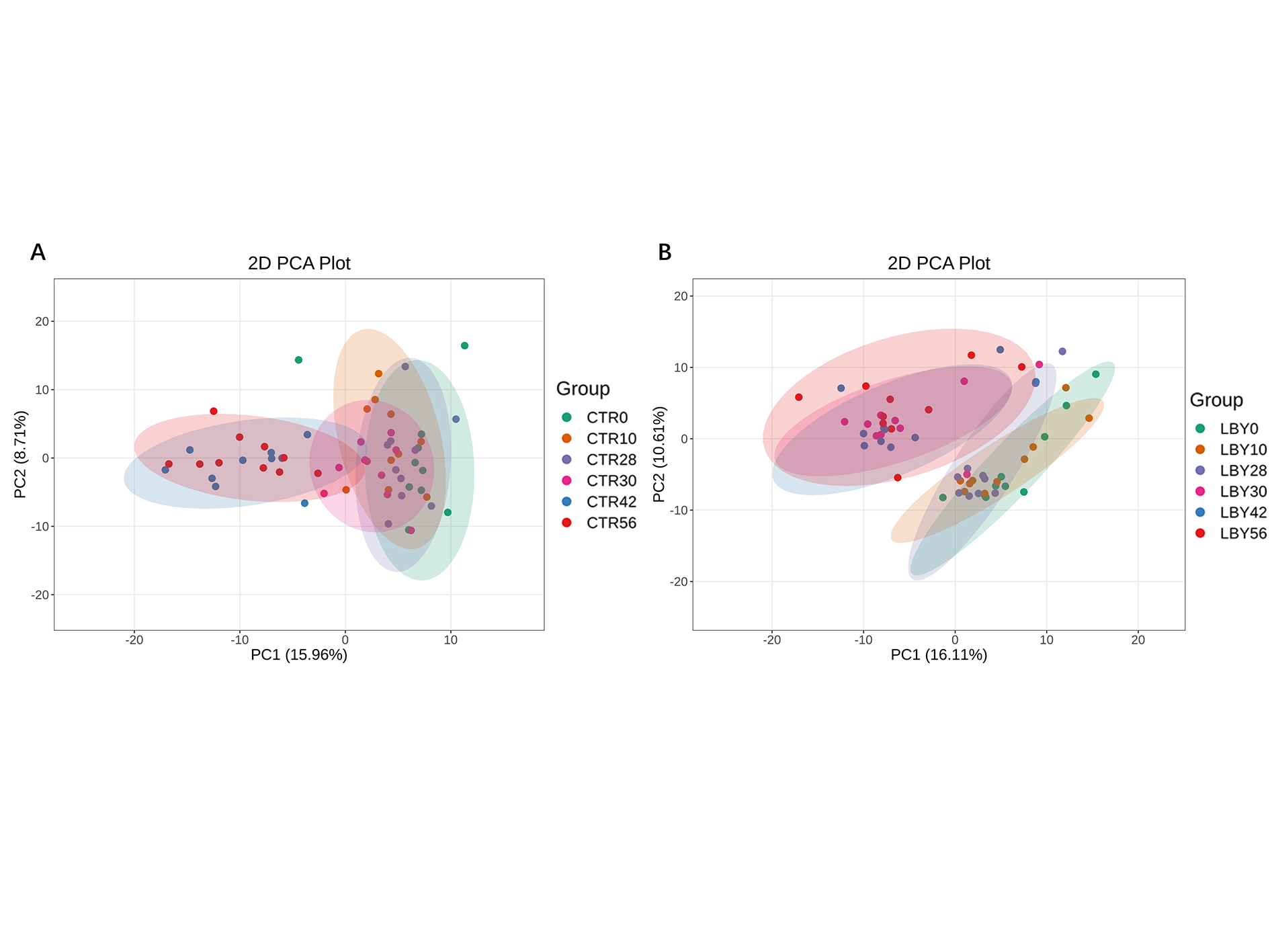


Supplementary Figure 3. Diet alters the gut metabolite. The PCA of CON (A) and YPS (B) groups. CON, control group; YPS, yeast probiotic supplementation.


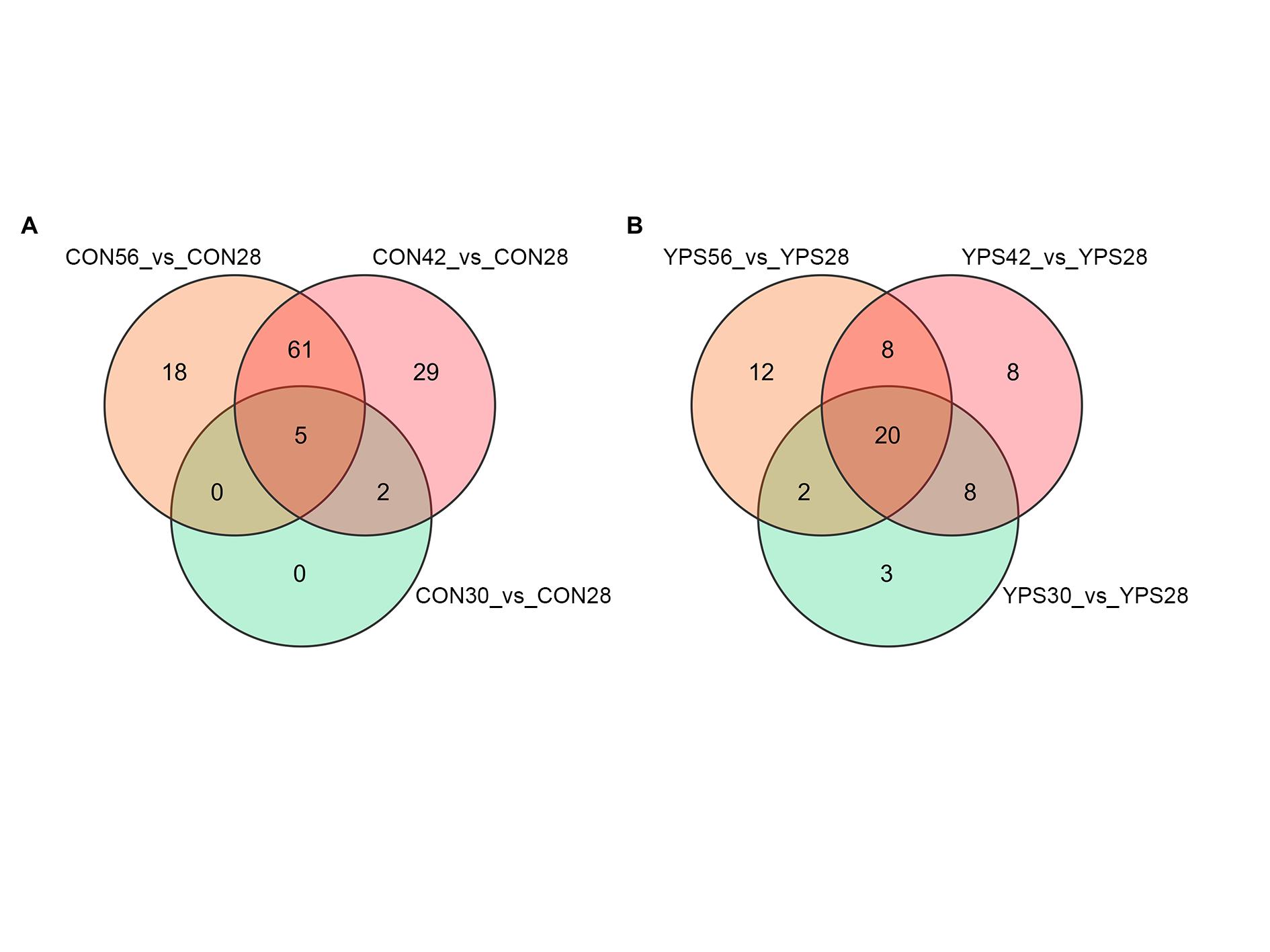


Supplementary Figure 4. Venn diagram summarizing the number of differential metabolites after diet transition in CON (A) and YPS (B) groups. CON, control group; YPS, yeast probiotic supplementation.


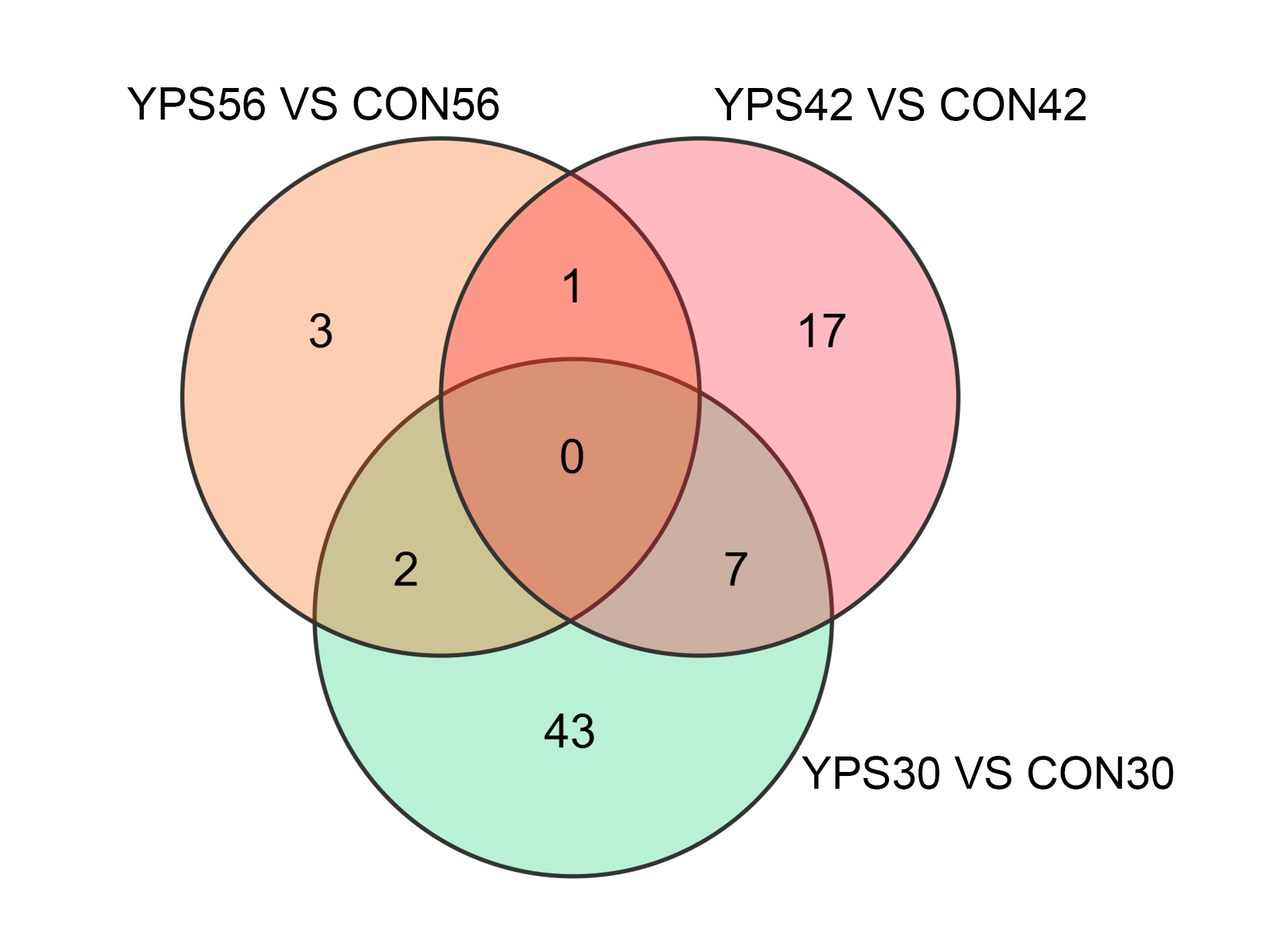


Supplementary Figure 5. The differential metabolites between CON and YPS groups after diet transition. CON, control group; YPS, yeast probiotic supplementation.
